# Analytic Tools for Post-traumatic Epileptogenesis Biomarker Search in Multimodal Dataset of an Animal Model and Human Patients

**DOI:** 10.3389/fninf.2018.00086

**Published:** 2018-12-20

**Authors:** Dominique Duncan, Giuseppe Barisano, Ryan Cabeen, Farshid Sepehrband, Rachael Garner, Adebayo Braimah, Paul Vespa, Asla Pitkänen, Meng Law, Arthur W. Toga

**Affiliations:** ^1^Laboratory of Neuro Imaging, USC Stevens Neuroimaging and Informatics Institute, Keck School of Medicine of USC, University of Southern California Los Angeles, CA, United States; ^2^Division of Neurosurgery, Department of Neurology, University of California at Los Angeles School of Medicine Los Angeles, CA, United States; ^3^A.I. Virtanen Institute for Molecular Sciences University of Eastern Finland, Kuopio, Finland

**Keywords:** MRI, EEG, epilepsy, epileptogenesis, informatics, neuroimaging, TBI, biomarker

## Abstract

Epilepsy is among the most common serious disabling disorders of the brain, and the global burden of epilepsy exerts a tremendous cost to society. Most people with epilepsy have acquired forms of the disorder, and the development of antiepileptogenic interventions could potentially prevent or cure epilepsy in many of them. However, the discovery of potential antiepileptogenic treatments and clinical validation would require a means to identify populations of patients at very high risk for epilepsy after a potential epileptogenic insult, to know when to treat and to document prevention or cure. A fundamental challenge in discovering biomarkers of epileptogenesis is that this process is likely multifactorial and crosses multiple modalities. Investigators must have access to a large number of high quality, well-curated data points and study subjects for biomarker signals to be detectable above the noise inherent in complex phenomena, such as epileptogenesis, traumatic brain injury (TBI), and conditions of data collection. Additionally, data generating and collecting sites are spread worldwide among different laboratories, clinical sites, heterogeneous data types, formats, and across multi-center preclinical trials. Before the data can even be analyzed, these data must be standardized. The Epilepsy Bioinformatics Study for Antiepileptogenic Therapy (EpiBioS4Rx) is a multi-center project with the overarching goal that epileptogenesis after TBI can be prevented with specific treatments. The identification of relevant biomarkers and performance of rigorous preclinical trials will permit the future design and performance of economically feasible full-scale clinical trials of antiepileptogenic therapies. We have been analyzing human data collected from UCLA and rat data collected from the University of Eastern Finland, both centers collecting data for EpiBioS4Rx, to identify biomarkers of epileptogenesis. Big data techniques and rigorous analysis are brought to longitudinal data collected from humans and an animal model of TBI, epilepsy, and their interaction. The prolonged continuous data streams of intracranial, cortical surface, and scalp EEG from humans and an animal model of epilepsy span months. By applying our innovative mathematical tools via supervised and unsupervised learning methods, we are able to subject a robust dataset to recently pioneered data analysis tools and visualize multivariable interactions with novel graphical methods.

## Introduction

The goal of the Epilepsy Bioinformatics Study for Antiepileptogenic Therapy (EpiBioS4Rx) is to identify relevant biomarkers of epileptogenesis after traumatic brain injury (TBI) and perform rigorous preclinical trials that permit the future design and performance of economically feasible full-scale clinical trials of antiepileptogenic therapies. Discovering these biomarkers of epileptogenesis is challenging, because this process is multifactorial and involves multiple modalities. We have been collecting and analyzing multimodal data, including neuroimaging, electrophysiology, and molecular/serological/tissue. An informatics infrastructure has been created to facilitate analysis and collaboration among scientists from various centers around the world (Duncan et al., 2018b). We have been developing innovative analytic tools to be shared with the broader epilepsy research community so that others may use our tools in addition to their own tools to advance research in this field. By working on this difficult problem collaboratively among researchers who possess different areas of expertise, we expect to identify several biomarkers of post-traumatic epileptogenesis from the multimodal data collected as part of EpiBioS4Rx and validate those biomarkers.

Substantial research has been devoted to investigate imaging biomarkers of epileptogenesis following TBI in an effort to better understand, prevent, and potentially treat post-traumatic epilepsy (PTE). Although incidence of PTE has been correlated with various factors, these results have been gathered and interpreted independently and are often drawn from models of human temporal lobe epilepsy, animal models of induced TBI via fluid percussion injury (FPI), and pilocarpine or kainic acid-induced status epilepticus. There has been limited investigation directly comparing these models to human cohort studies of epileptogenesis following trauma, which is one area in which our work extends on existing research on PTE. Also, few multimodality studies have been conducted to investigate interrelations among identified potential biomarkers, which could assist in establishing a panel of non-invasive epileptogenic biomarkers that consistently precedes and predicts the development of PTE. EpiBioS4Rx is collecting large-scale imaging data on TBI patients with subsequent seizure activity as well as imaging data on a rodent model of TBI, allowing for a multimodality and multi-species investigation.

Several reviews have summarized electrophysiological (Worrell, 2011; Staba et al., 2014) and imaging (Mishra et al., 2011; van Vliet et al., 2017; Pitkänen et al., 2018) biomarkers identified in rat models and human patients in recent years. Notably, high frequency oscillations (HFOs), standard frequency between 80 and 600 Hz (Staba et al., 2014), are consistently produced by epileptic neural tissues (Bragin Engel et al., 1999; Jacobs et al., 2012; Zijlmans et al., 2012) and have also been reported in rats after administration of lateral FPI within or adjacent to the injured tissue (Reid et al., 2016). In the same FPI model, pathologic HFOs and repetitive HFOs and spikes (rHFOSs) occurred within 2 weeks of insult only in rats that would later develop seizures (Reid et al., 2016). However, currently there are no validated electrophysiological biomarkers of post-traumatic epileptogenesis (Perucca et al., 2018), so one of our goals is to identify electrophysiological biomarkers that can be validated. As many models of PTE involve continuous EEG recordings, automated seizure detection programs have been investigated to ease data analysis. Approximate entropy (ApEn), in conjunction with neural networks, has been introduced as an analytic tool to discriminate normal and ictal or pre-ictal EEG from epileptic patients and healthy controls (Liang et al., 2010), refining and enhancing seizure detection, which can ultimately expedite the EEG analysis workflow.

Magnetic Resonance Imaging (MRI) and Diffusion Tensor Imaging (DTI) have allowed for non-invasive analysis of molecular and structural alterations of white matter and other neural structures at high spatial resolution. MRI may be used to identify specific abnormalities associated with increased susceptibility to epileptogenesis, including focal lesions (D'Alessandro et al., 1982; Dalessandro et al., 1988), intracerebral hemorrhage (D'Alessandro et al., 1982), biparietal contusions (Englander et al., 2003) and dural penetration from bone or metal fragments (Englander et al., 2003). In a lateral FPI model, diffusion tensor trace alterations in the hippocampus acquired 3 hours after injury were found to predict seizure susceptibility and number of spikes 12 months later (Kharatishvili et al., 2007). A follow up study confirmed that Dav (one third of the trace of the diffusion tensor that is an orientation-independent measure of water diffusion) at 23 days and 2 months and T1p (a longitudinal relaxation in the rotating frame, which can be assumed to be similar to T1 relaxation in the very low magnetic field, thus probing interaction between water and macromolecules in the tissue) at 9 days post insult could predict increased seizure susceptibility following lateral FPI (Immonen et al., 2013).

Axonal damage, visualized with DTI, is seen across all severities of TBI, although irreversible myelin damage, which is correlated with worse cognitive prognoses, is more typically caused by moderate and severe TBI (Kraus et al., 2007). Decreased fractional anisotropy (FA) has been repeatedly found in TBI patients compared with healthy controls (Bendlin et al., 2008; Sidaros et al., 2008; Irimia et al., 2014), which is especially relevant considering FA ratios have been found to be significantly reduced in TBI patients who developed late post-traumatic seizures compared with non-epileptic TBI patients (Gupta et al., 2005), and along temporal lobe white matter in benign mesial TLE (Labate et al., 2015). Additionally, connectomic studies and tract-based spatial statistics may assist in the understanding of how white matter degeneration patterns lead to neural and cognitive impairment (Irimia et al., 2014), so they may also support a greater understanding in how degeneration patterns specifically lead to PTE. We plan to use our pipelines for connectomics to understand the development of PTE as well as relate these imaging data to the electrophysiological data.

MRI also serves as a useful tool for morphometric analysis. TBI varies significantly in the severity of insult and subsequent lesion(s), so precise lesion quantification is necessary to compare outcomes following stratified severity of injury. Voxel-based morphometry analysis has indicated reduced hippocampal and thalamic volumes in TLE patients (Labate et al., 2008). In a lateral FPI model, Shultz et al. found that hippocampal surface shape analysis (conducted via MRI-based large-deformation high-dimensional mapping) at 1 week post-injury could be predictive of PTE. Rats that later developed PTE showed increased lateral regions while non-epileptic rats showed decreased medial and ventral regions (Shultz et al., 2013). We have developed analysis pipelines to analyze both animal and human imaging data to relate these and explore the translational components of the animal data.

Several supervised and unsupervised models of lesion identification and quantification from T1, T2, and FLAIR images acquired from MRI have been introduced in an effort to automate analysis of multiple sclerosis (Wetter et al., 2016), tumor (Guo et al., 2015), chronic stroke (Pustina et al., 2016; Guo et al., 2018), and TBI (Irimia et al., 2011). Automated quantification of TBI lesions by normalizing and standardizing against standard templates is challenging given that brain morphology is often distorted due to insult (Kim et al., 2008), so our work aims to quantify TBI lesions automatically while maintaining accuracy.

Transforming Research and Clinical Knowledge in TBI (TRACK-TBI) was a study performed at the University of California, San Francisco (main site) that proved the feasibility of large-scale, multi-site analysis of imaging, blood, and clinical data on nearly 3,000 TBI patients. Patient data gathered through TRACK-TBI have been used to examine the relationship between CT and MRI findings that are commonly assessed in emergency trauma facilities and DTI, both of which have been reported as potential biomarkers of epileptogenesis following TBI. In mild TBI cases, FA is significantly reduced in CT/MRI-positive (acute intracranial lesion, including epidural or subdural hematoma, subarachnoid hemorrhage, contusion, axonal injury, or skull fracture) and not reduced in CT/MRI-negative patients (Yuh et al., 2014). DTI can detect alterations in microstructural white matter with greater subtlety than MRI, and FA ratios have been found to be significantly reduced in TBI patients who developed late PTS compared with non-epileptic TBI patients (Gupta et al., 2005). In another study, mild TBI patients with CT/MRI-positive (defined as having any evidence of lesion) and CT/MRI-negative (no lesions) showed distinct alterations of functional connectivity in resting state fMRI analysis within days of injury that were predictive of cognitive outcomes 6 months later (Palacios et al., 2017).

The EpiBioS4Rx informatics infrastructure contains a thorough and harmonized multimodal database, including imaging and EEG data, which enables researchers to correlate results from imaging analysis to longitudinal epileptiform activity (Duncan et al., 2018b) from both humans and an animal model. Recently, analysis of EpiBioS4Rx data found that early post-traumatic seizures and subsequent development of PTE following severe TBI are strongly correlated with lesions localized to the temporal lobe (i.e., hemorrhagic temporal lobe injury) but not general lesion severity (as measured by the Glasgow Coma Scale) (Tubi et al., 2018).

## Data

The total amount of data that has been and will be collected in the ongoing EpiBioS4Rx includes EEG and video-EEG (video tape recording during EEG monitoring) from cohorts of animals after TBI (using FPI) recorded continuously for 6 months, in addition to prolonged continuous intensive care unit (ICU) EEG recordings from 300 humans, including depth EEG from 100 patients, and intermittent sampling of brain images, blood, and tissue data over 2 years. The collected rat MRI consist of structural and diffusion weighted measures. Sprague-Dawley control rats and TBI rats (left lateral fluid percussion injury) were used with data collected using a Bruker BioSpin MRI GmbH using a dtiEpiT SpinEcho sequence (Duncan et al., 2018b). Patients admitted into the ICU after an acute moderate-severe TBI involving a frontal and/or temporal lobe hemorrhagic contusion are screened for the study. Although a number of sites are collecting data for EpiBioS4Rx, we focus our preliminary analysis on human data from the University of California, Los Angeles (UCLA) and animal data from the University of Eastern Finland, Kuopio.

## Analysis Methods

We present a collection of analytic tools for this multimodal dataset and present examples of some preliminary work on sample data from EpiBioS4Rx as well as future directions for this analysis.

### Imaging Methods

We have developed a multimodal image analysis workflow that includes lesion mapping and tractography reconstruction of white matter pathways. Additionally, we have analyzed paravascular spaces (PVS) in the MRI data to aid in our search for post-traumatic epileptogenesis biomarkers.

#### Lesion Mapping

**Figure 1:**
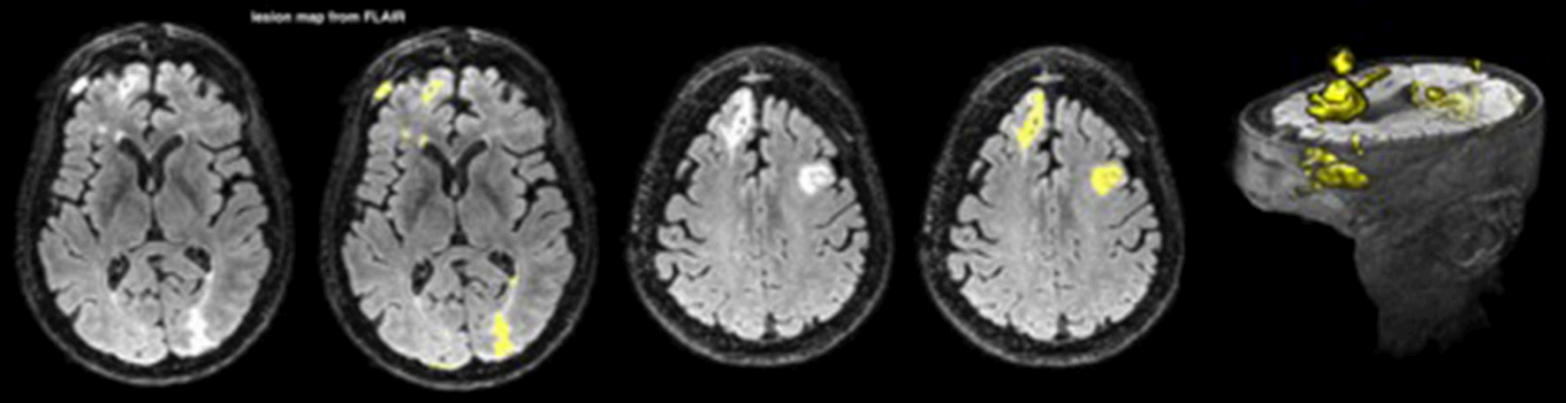
A lesion map from FLAIR for one patient; the lesions are depicted in yellow.

Lesions were mapped from fluid-attenuated inversion recovery (FLAIR) images with an automated segmentation pipeline using FMRIB Software Library (FSL) tools (Woolrich et al., 2009; Jenkinson et al., 2012; Wetter et al., 2016). FLAIR suppresses the signal produced by cerebrospinal fluid (CSF) and is sensitive to contrast for mapping lesions in TBI (Gentry et al., 1988; Bigler, 2001; Narayana, 2017). The pipeline begins with skull stripping, smoothing, and intensity normalization. Then lesions are separated from brain tissue and CSF using a histogram-based thresholding algorithm. Finally, lesions not overlapping with white matter (WM) are discarded by registering a WM mask from a standard space into the subject space (FSL-FNIRT is used for the registration). An example is shown in Figure 1.

In order to separate periventricular WM hyperintensities from the rest of the WM lesions, we performed a secondary analysis on the T1-weighted (T1w) images. Structural T1w images are less sensitive to periventricular lesions due to CSF partial volume effect, yet they can visualize WM lesions across the brain. The T1w images were analyzed through a similar pipeline as the FLAIR images, and the lesions were mapped accordingly.

#### Tractography

**Figure 2:**
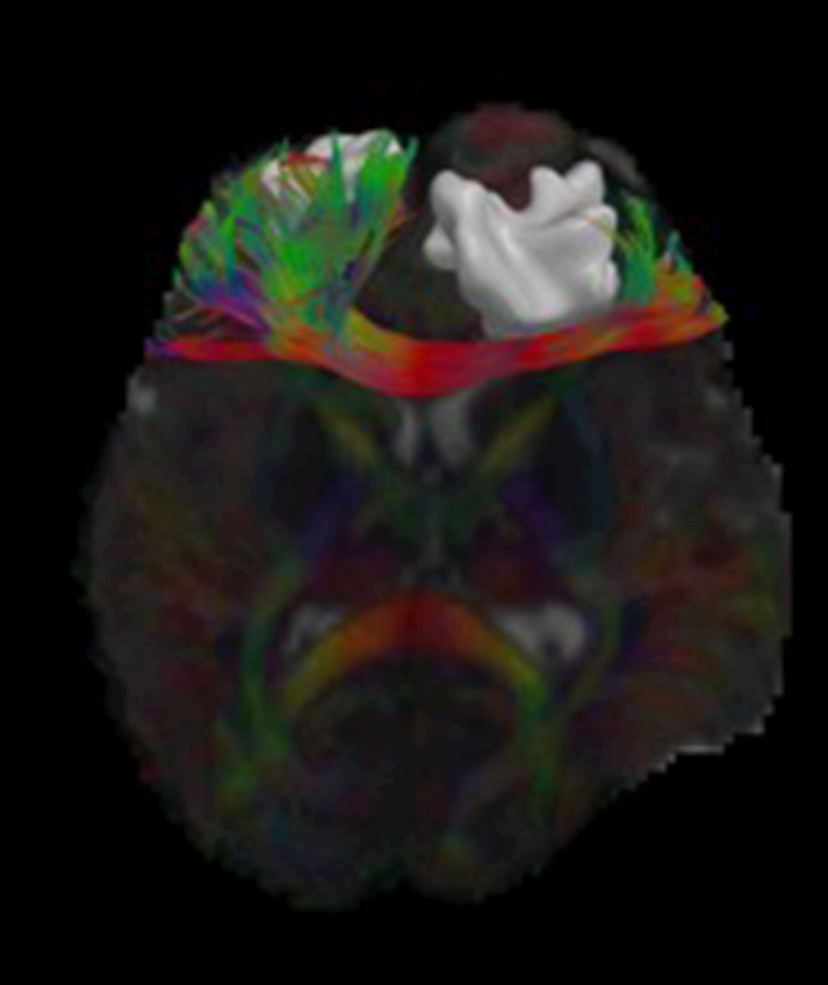
Visualizations of diffusion MRI data from a single human subject. The image shows an axial brain slice rendered with glyphs depicting the underlying multi-compartment diffusion models. A tractography reconstruction of the forceps minor is shown alongside a brain lesion. Through 3D modeling and visualization, we are able to show the impact of the brain trauma on structural connectivity of the frontal lobe.

**Figure 3:**
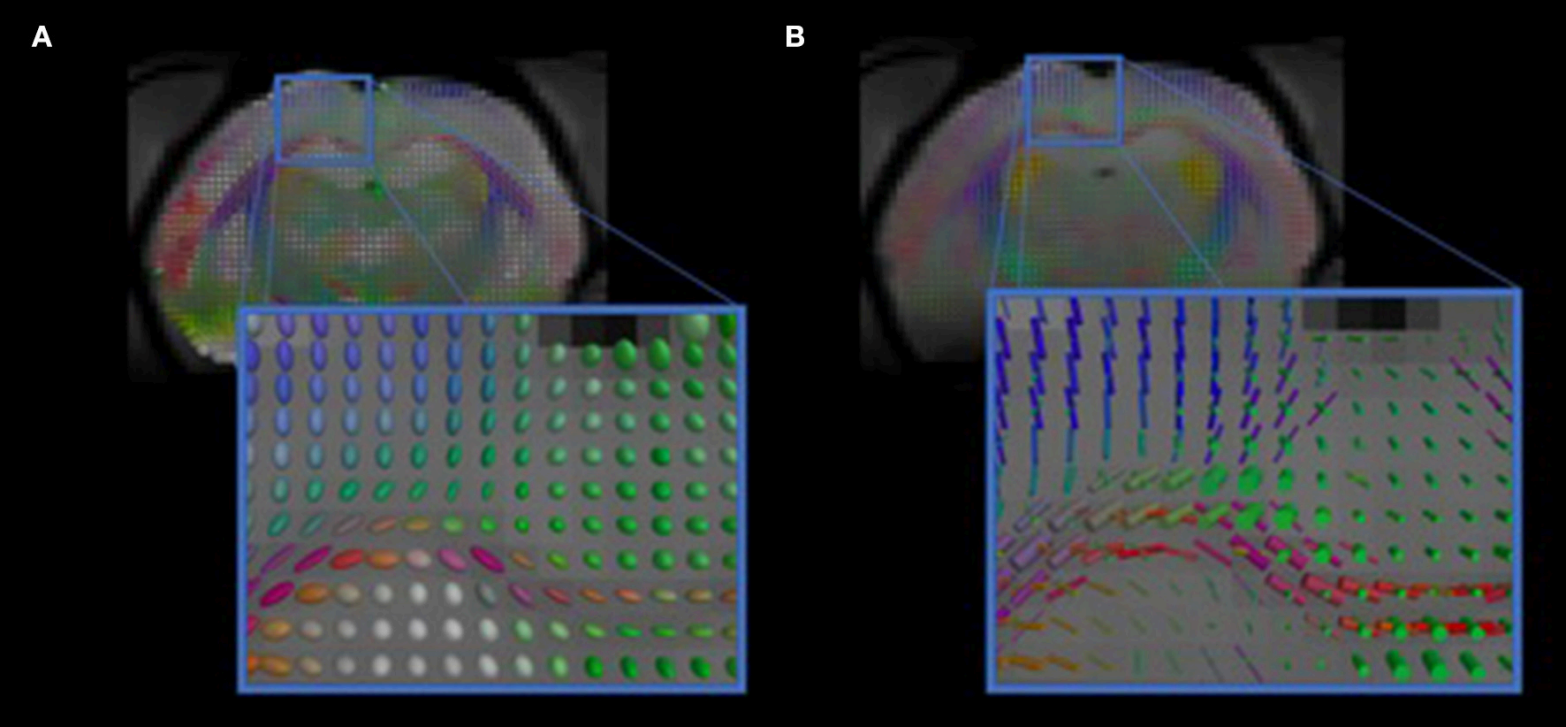
Visualizations of diffusion MRI from the rodent data. The images show diffusion models estimated in each voxel. **(A)** shows standard diffusion tensor modeling, and **(B)** shows multi-compartment modeling that resolves complex anatomical features, such as crossing fibers.

**Figure 4:**
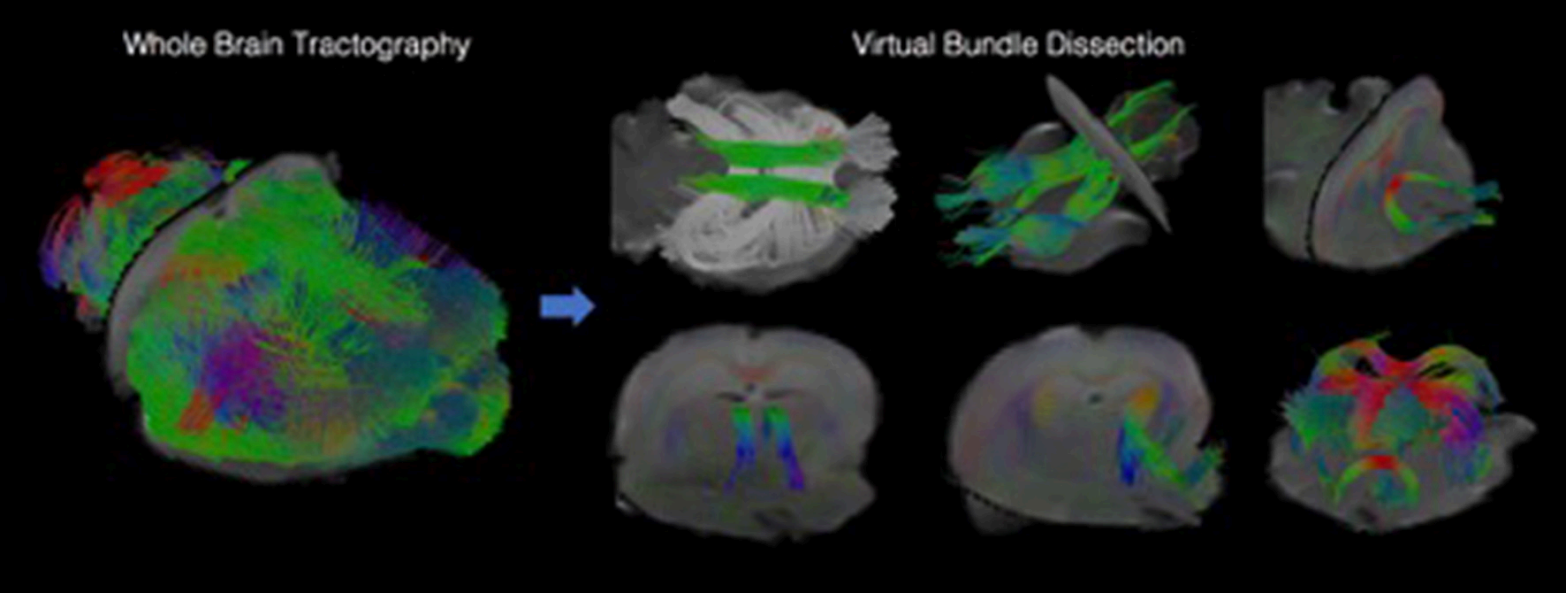
Visualizations showing tractography-based modeling of rodent imaging data. Multi-fiber tractography was used to create geometric models depicting the trajectory of white matter fiber bundles. The left panel shows results from whole brain tractography, and the right panel shows how whole brain results can be decomposed into specific fiber bundles using virtual dissection.

We have developed diffusion MR image analysis pipelines for quantitative analysis of WM microstructure and connectivity across both rodent and human datasets. Tractography models were created from the diffusion-weighted MRI (dMRI) data using FSL (Jenkinson et al., 2012) and the Quantitative Imaging Toolkit (QIT) (Cabeen et al., 2018). The dMRIs were first skull stripped using FSL Brain Extraction Tool (BET) and then corrected for motion and eddy current artifacts using FSL FMRIB's Linear Image Registration Tool (FLIRT). For this, each diffusion scan was affinely registered to the baseline scan using the mutual information metric, and the associated gradient orientations were rotated to account for the registration. Diffusion tensor models were then estimated from the dMRI using QIT, and the following tensor parameters were extracted: fractional anisotropy (FA), mean diffusivity (MD), axial diffusivity (AD), and radial diffusivity (RD). A study specific template was created using Diffusion Tensor Imaging ToolKit (DTI-TK) (Zhang et al., 2006), and the deformation field for each scan was used to register the data to the Illinois Institute of Technology (IIT) brain template (Zhang et al., 2011) to subject native space. Tractography models of our bundles of interest, including uncinate fasciculus, anterior thalamic radiation, corticospinal tract, inferior longitudinal fasciculus, superior longitudinal fasciculus, fornix, arcuate fasciculus, and five subdivisions of the corpus callosum were created using a framework for deterministic streamline integration (Cabeen et al., 2016). For each bundle, seed, inclusion, and exclusion masks were manually drawn in the IIT template (Wakana et al., 2007) in reference to a white matter atlas (Catani and Thiebaut de Schotten, 2008). The template masks were then resampled in each subject's native space image to constrain tractography. Other tractography parameters included a step size of 1.0 mm, a maximum angle of 45°C, and a minimum FA of 0.15–25,000 seeds per bundle. Bundle-specific metrics were then computed, including bundle volume, track density, track length, and averages of DTI metrics listed above. In addition to tractography analysis, the human data were also analyzed using voxel-based analysis to obtain diffusion MRI metrics in anatomical regions derived from the Johns Hopkins white matter atlas (Mori et al., 2008; Cabeen et al., 2017). This method applies to human data (Figure 2) as well as rodent data (Figures 3, 4). We found that the data allowed multi-fiber modeling to resolve partial volume effects and crossing fiber configurations.

#### Paravascular Spaces

Many studies have shown that paravascular spaces (PVSs) may play an important role in neuroinflammation: a strong post-traumatic inflammatory reaction was documented in PVSs of contused human brain tissue, suggesting that PVSs' impairment could explain the altered macrophage activity resulting in seizure onset (Holmin et al., 1998; Bechmann et al., 2001; Corraliza, 2014; Abiega et al., 2016). Also structural changes in PVSs may affect their surrounding white matter networks (Taoka et al., 2017). We investigate the role of paravascular spaces in TBI as a potential biomarker for post-traumatic epilepsy.

##### Study population

We present some analysis performed on human data, focusing on PVSs' role as a potential biomarker of epileptogenesis after TBI; we analyzed clinical data and MRI scans in a sample of 15 patients (12 males, 3 females, age range: 7–68 years old). MRI scans were performed 14 days after trauma using a 3T MRI scanner. PVSs were analyzed on 3D T2 Turbo Spin Echo (TSE) sequences. Six healthy subjects (3 males, 3 females, age range: 12–62 years old) were used as controls. Demographic characteristics of TBI patients and healthy subjects are summarized in Table 1.

**Table 1:** Demographic characteristics of TBI patients and healthy subjects.

**Characteristics**		**TBI patients**	**Healthy controls**
Subjects (#)	Total	15	6
	Male	12	3
	Female	3	3
Age at scan (years)[Mean ± standard
deviation]	Total	34 ± 23	30 ± 17
	Male	35 ± 22	23 ± 12
	Female	30 ± 29	37 ± 22

##### PVS analysis

PVSs were defined as tubular-linear or round-ovoid structures with a CSF-like signal intensity (hyperintense on T2-weighted images) and a diameter of < 3 mm. PVSs surround perforating vessels in the brain, and the largest number of PVSs is usually found in the basal ganglia and centrum semiovale. The typical shape, dimensions, and location were used to exclude other possible differential diagnoses (e.g., lacunar infarcts). In this study, we omitted PVS with a diameter of < 0.5 mm, because their identification and measurement were not sufficiently reliable.

Image processing on the 3D T2 TSE images was performed in OsiriX Image Viewing Software (Ratib and Rosset, 2006) by a reader blinded to subjects' clinical data. In each subject, we manually marked and counted all PVSs with a diameter between 0.5 and 3 mm. The caliber of PVS was measured with the Ruler Tool in OsiriX. Both the total number of PVSs and the caliber of each PVS were systematically recorded. We categorized PVS by location in the cerebral hemisphere (right and left) to assess the distribution of PVS in the brain. Because of the possible inter-individual variability in the total number of PVSs, we calculated 2 ratios (*HR*_*righ*__t_ and *HR*_*left*_) between each hemisphere's amount of PVS (*PVS*_*right*_ and *PVS*_*left*_, respectively) and the sum of PVS in the whole brain (*PVS*_*tot*_) for each subject:

Two possible outcomes resulted from these ratios:

- Two equivalent values (*HR*_*right*_ = *HR*_*left*_ = 0.5) if no difference in the number of PVSs was found between the right and left hemispheres;
- Two different values (*HR*_*right*_ ≠ *HR*_*left*_) if the number of PVSs was not the same in the 2 hemispheres. In this situation, we defined *HR*_*minor*_ and *HR*_*major*_ to be the ratios obtained from the hemisphere with less and more PVS compared to the contralateral hemisphere, respectively. Consequently:

(1) $$\begin{array}{l} 0.0<HR_{minor}<0.5\;and\;0.5>HR_{major}>1 \end{array}$$

Then we calculated the difference between *HR*_*minor*_ and *HR*_*major*_ as an asymmetry index (AI):

(2) $$\begin{array}{l} \text{AI}=\text{HRmajor}-\text{HRminor} \end{array}$$

with 0 ≤ AI ≤ 1

The higher the AI value was, the more asymmetric the distribution of PVS in the brain was. As a physiological right-left asymmetry in the brain has been reported in previous studies (Asgari et al., 2016; Feldman et al., 2018), and an unbalanced distribution of PVS may be considered normal, we used a threshold of AI ≥ 0.2 to define a significantly high asymmetry in PVS distribution. This value means that one hemisphere has more than 60% of the total number of PVSs.

We measured the caliber of each marked PVS, and the average of PVS caliber in the right and left hemispheres (*C*_*right*_ and *C*_*left*_, respectively) in all subjects. Then we calculated the difference (*|C*_*diff*_*|*) between the mean PVS caliber in the two hemispheres:

(3) $$\begin{array}{l} |\text{Cdiff}|=\text{Cright}-\text{Cleft} \end{array}$$

##### Statistical analysis

A Student's *t*-test was used to determine if there was a difference in the total number and the mean distribution of PVSs between the two cerebral hemispheres in the healthy controls and TBI group. A difference of *p* < 0.05 was considered statistically significant.

## Results

### Total Number of PVSs

We evaluated the total number of PVSs in TBI patients and healthy controls: the average was 77 ± 48 in the first group, and 80 ± 15 in the latter. No significant difference was found between the two groups (*p* = 0.40).

**Figure 5:**
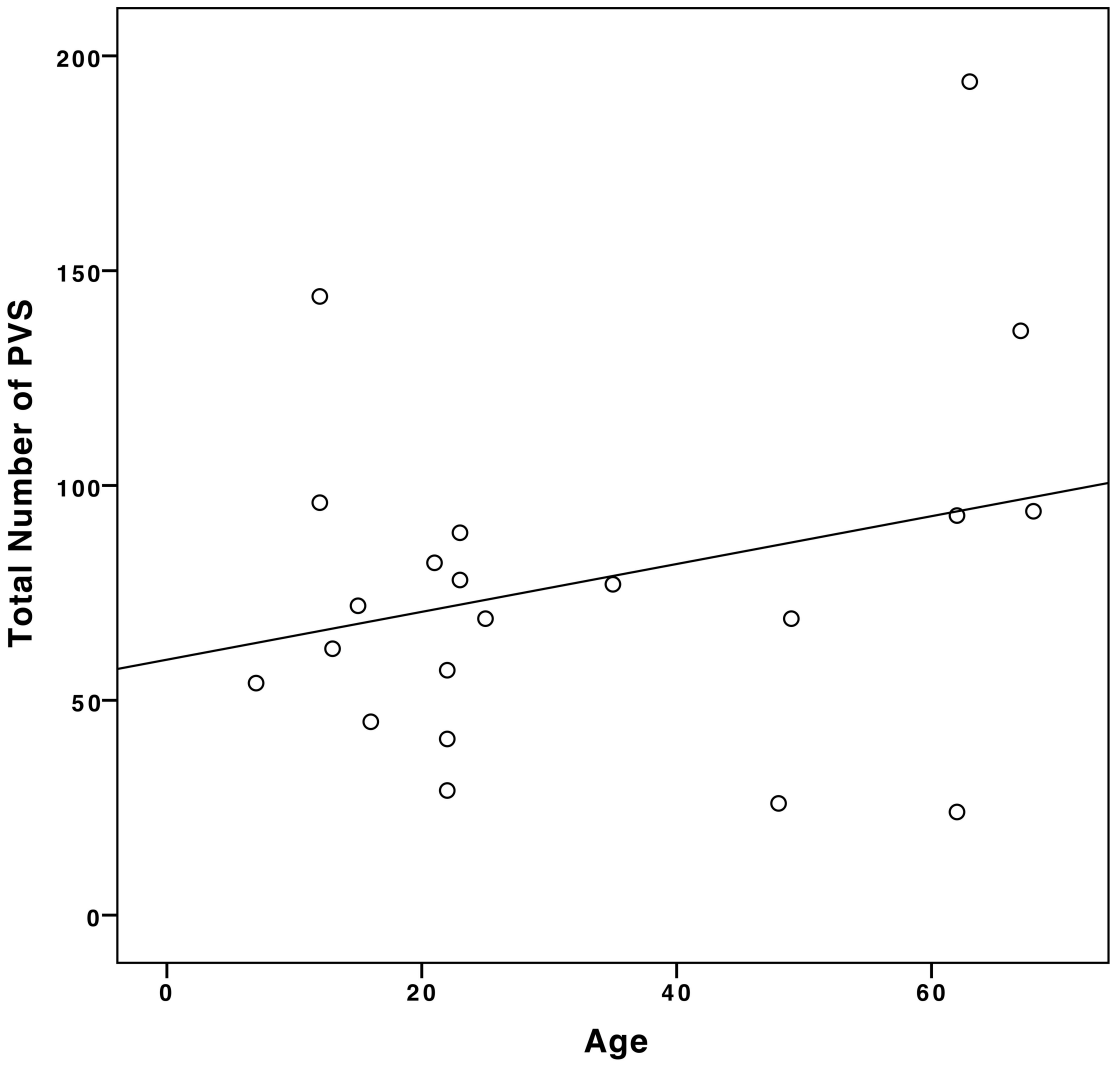
Correlation between age and the number of PVSs in our sample population.

In our population, we found a weak positive correlation between age and the number of PVSs (Pearson's ρ = 0.28, *p* = 0.11), as shown in Figure 5.

### Asymmetry Analysis

**Figure 6:**
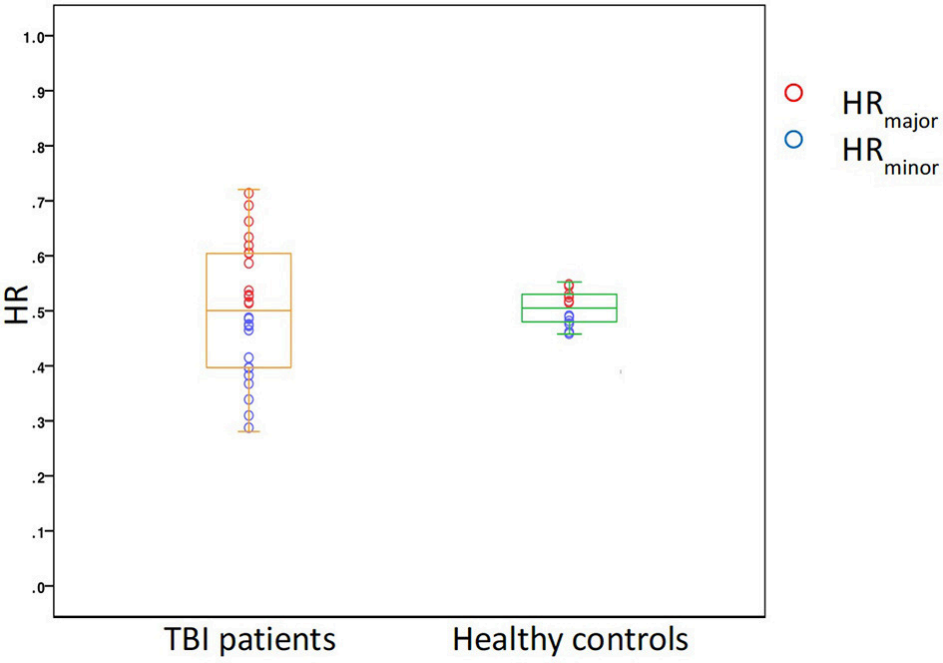
A box plot showing the distribution of HR in the two study groups, TBI patients and healthy controls.

Both TBI patients and healthy controls presented a different number of PVSs in the two cerebral hemispheres. The HR range was 0.29–0.71 in TBI patients and 0.43–0.54 in healthy controls; in the patient group, the mean *HR*_*minor*_ and *HR*_*major*_ were 0.42 and 0.58, respectively, while in the control group, the values were 0.48 and 0.52, respectively (Figure 6). The degree of asymmetry was significantly different in the two groups (*p* = 0.001): the average AI was 0.17 in TBI patients and 0.04 in control subjects.

**Figure 7:**
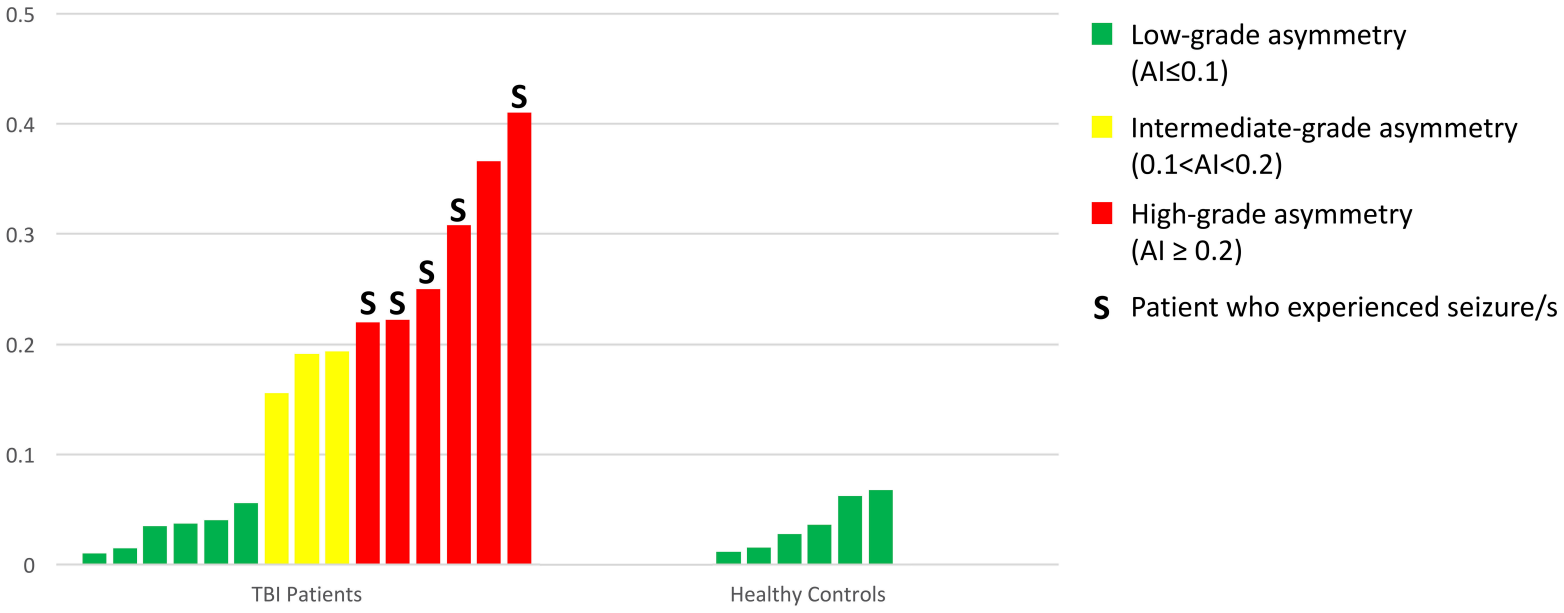
A bar graph showing all AIs in the 15 TBI patients and 6 healthy controls.

In the TBI group, we found six patients with a highly asymmetric distribution of PVS (Figure 7) in the two cerebral hemispheres (AI ≥ 0.2). Five of these patients (83%) experienced at least one seizure within the first six months after TBI (in four cases, the seizure happened within the first month); in three cases, Lateralized Periodic Discharges (LPDs) were detected in the EEG, and in all cases, the affected hemisphere matched with the hemisphere where less PVSs were identified. Furthermore, in all nine TBI patients with intermediate- or high-grade PVS asymmetry, the cerebral hemisphere that suffered the trauma showed a minor number of PVSs compared with the contralateral side.

### PVS Caliber Analysis

**Figure 8:**
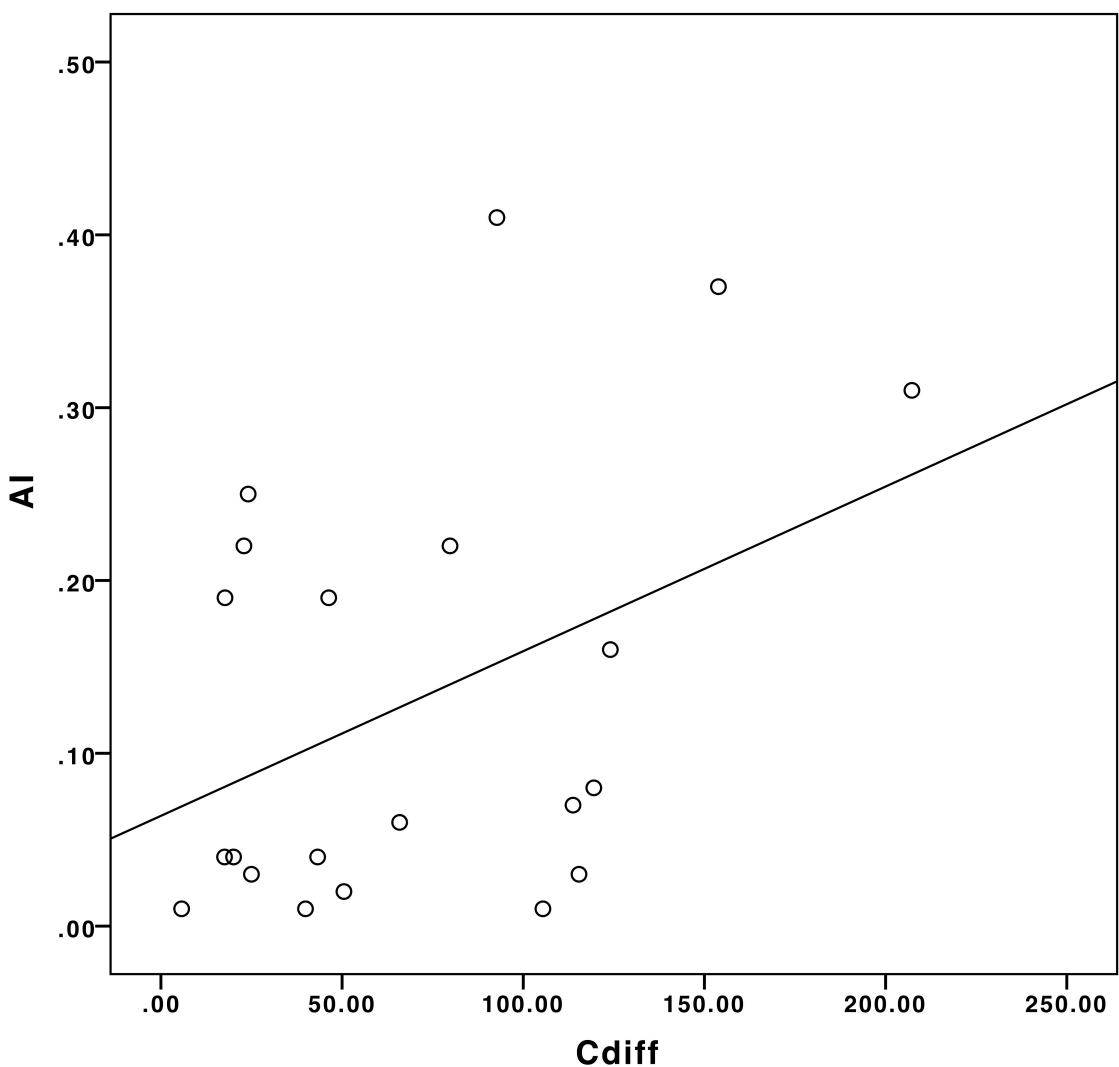
The correlation between C_*diff*_ and AI in our sample population.

The mean PVS caliber in TBI patients and healthy controls were 1.37 ± 0.23 mm and 1.31 ± 0.26 mm, respectively: the difference in the two groups was not statistically significant (*p* = 0.39). We found a significant positive correlation between *AI* and *|C*_*diff*_*|*, as illustrated in the scatter plot in Figure 8 (Pearson's ρ = 0.41, *p* = 0.03).

Patients with a more asymmetric distribution of PVS in the brain had a greater difference in the mean PVS caliber between right and left hemispheres. In patients who had a post-traumatic seizure, smaller PVSs were measured on the side ipsilateral to LPDs and/or affected by the trauma, compared with the contralateral hemisphere. In four patients, the difference in the PVS caliber between the two hemispheres was statistically significant (*p*-values were 0.031, 0.036, 0.034, and 0.049). Thus, the evaluation of PVS distribution and quantification may represent another potential non-invasive neuroimaging biomarker to predict the development of epilepsy after TBI.

### EEG Methods

Various analytic tools were used to analyze both human and rodent EEG. Notably, dimensionality reduction techniques, including diffusion maps and Unsupervised Diffusion Component Analysis (UDCA), were used to elucidate patterns or abnormal activity within large data matrices that may be used to potentially identify biomarkers of epileptogenesis after TBI. Spectral analysis and measures of relationship, such as mutual information, were also conducted. We present an overview of a few analytic tools for EEG with some figures of examples of preliminary results using EpiBioS4Rx data.

#### Spectral Analysis

**Figure 9:**
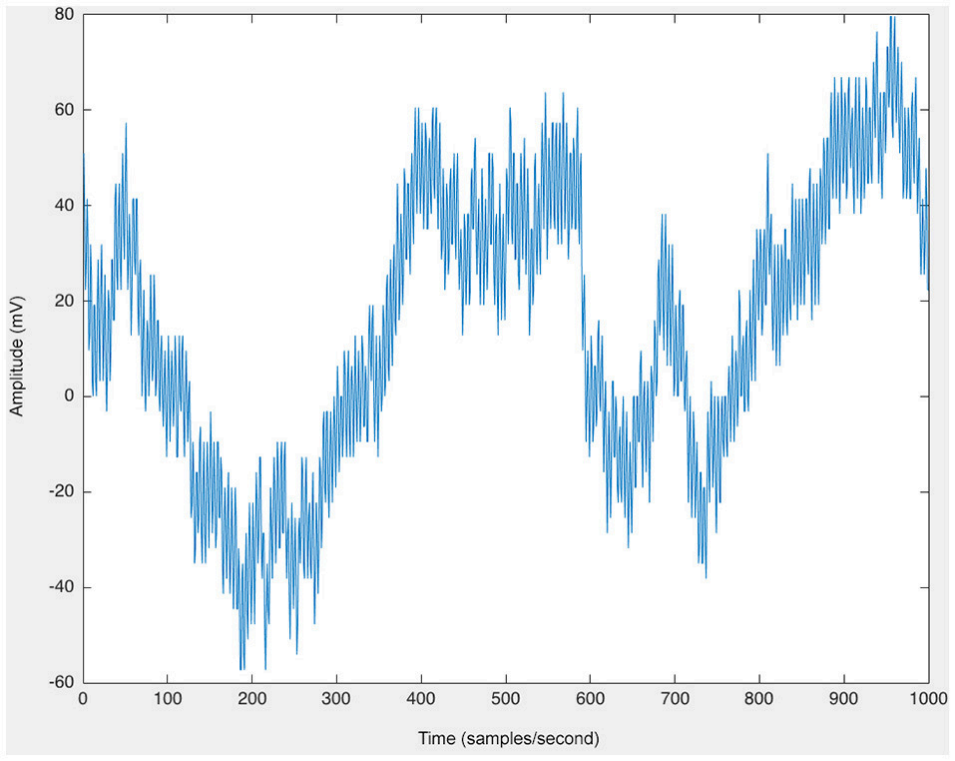
The raw EEG from one channel of human scalp EEG data (200 samples/second).

**Figure 10:**
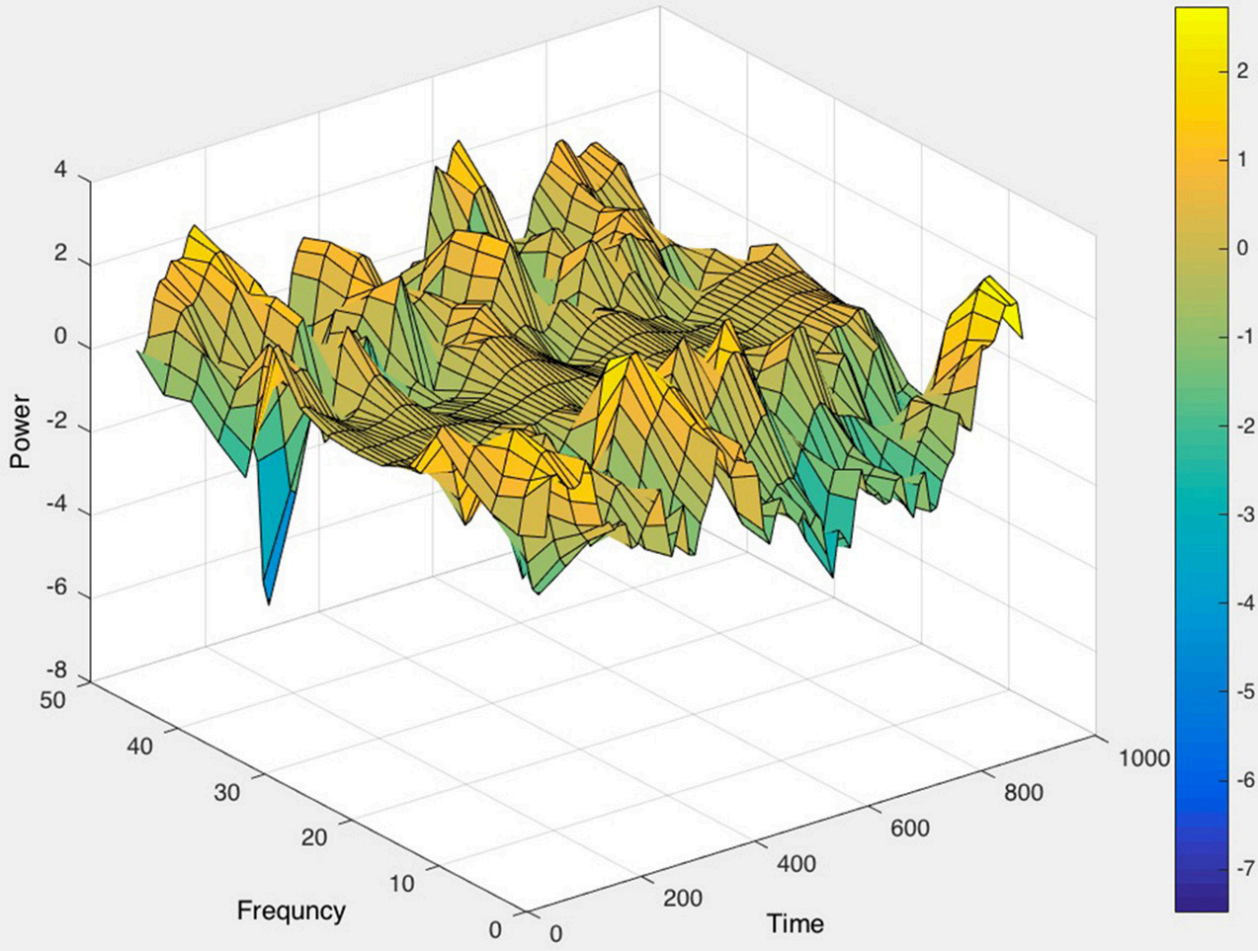
The 3D power spectral density (PSD), corresponding to the raw EEG data in Figure 9, displayed (in color) as well as the relationships among time, frequency, and magnitude power for a one-channel, brief sample segment of EEG data (200 samples/second).

As a first step, raw EEG data were imported via EEGLAB in MATLAB (Delorme and Makeig, 2004). The Short Time Fourier Transform (STFT) was applied to the raw, unfiltered EEG data, seen in Figure 9, and spectrograms were formed to visualize frequency changes over time. 3D spectrograms, such as Figure 10, show the relationship among time, amplitude, and power in addition to the power spectral density (PSD). These plots can be used for visualization purposes or for setting a threshold to focus on a specific frequency range, for example, and then quantifying changes over time.

#### Persyst Software Tools

We also use Persyst software (Sierra-Marcos et al., 2015) as a tool for visualization of the EEG and for artifact removal, spike detection, and epileptiform activity identification.

#### Mutual Information

Another type of analysis that we perform considers measures of relationship, such as mutual information (Duncan et al., 2013a), to study how electrical activity from different areas of the brain relate to each other and how those relationships change over time. We plan to relate these measures of relationship in the EEG to the resting state fMRI to determine if electrode contacts from areas within resting state networks have higher values of mutual information and if these networks differ between patients who develop PTE and those who do not.

**Figure 11:**
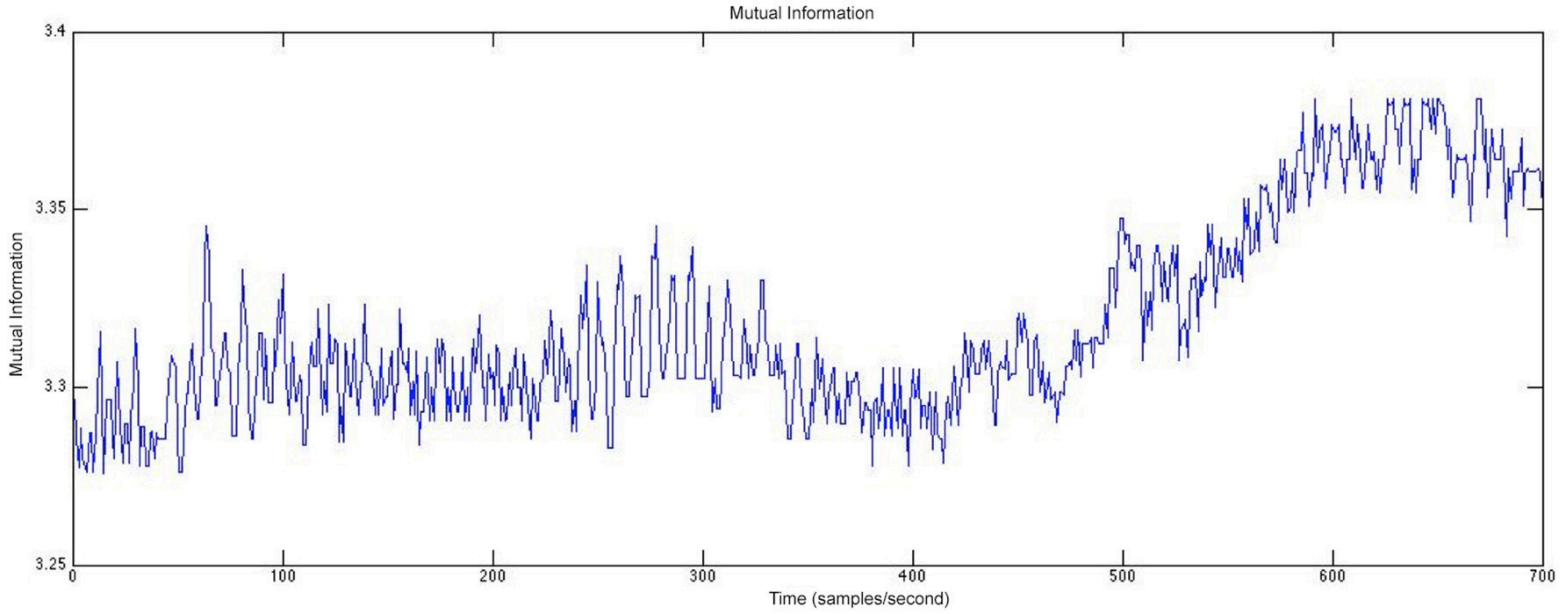
The mutual information between two channels calculated at 30-second windows of time for rodent EEG data.

Figure 11 shows an example of the mutual information between two channels of rodent EEG. The mutual information between the two channels was calculated for each consecutive 30-second window and plotted to visualize the relationship between the two channels located in different parts of the brain. This analysis allows us to study how this relationship changes both over time and closer to the occurrence of a seizure, which enables the study of networks in the brain and if those play a role in post-traumatic epileptogenesis. In Figure 11, we see a greater relationship between the two electrode contacts chosen for the analysis over time and closer to the seizure onset. Furthermore, we can compare these networks in rats and humans to determine the extent of their similarities.

#### Dimensionality Reduction

Besides analyzing EEG using spectral analysis, spike detection, and measures of relationship, we can also use dimensionality reduction techniques to analyse the data more extensively and classify epileptiform activity. The EEG amounts to a very large dataset due to the continuous long-term recordings over many electrode contacts. All 300 patients receive 24 h continuous EEG (cEEG) for 72 h minimum during the first 7 days after TBI. Scalp cEEG monitoring is performed using a 16–21 channel bipolar and referential composite montage implemented at each study center based on their established ICU EEG protocols. A subset of 100 patients receive additional depth EEG monitoring during the first 7 days after TBI for higher resolution and pathologic HFOs or repetitive HFOs and spikes detection. Furthermore, we have continuous EEG recordings over 6 months from many cohorts of animals (Duncan et al., 2018b).

An algorithm that we have developed, UDCA (Duncan and Strohmer, 2016; Duncan et al., 2018a), is an extension of diffusion maps (Coifman and Lafon, 2006) and used to reduce the dimensionality of this large amount of data as well as identify patterns in the data that may predict post-traumatic epileptogenesis.

**Figure 12:**
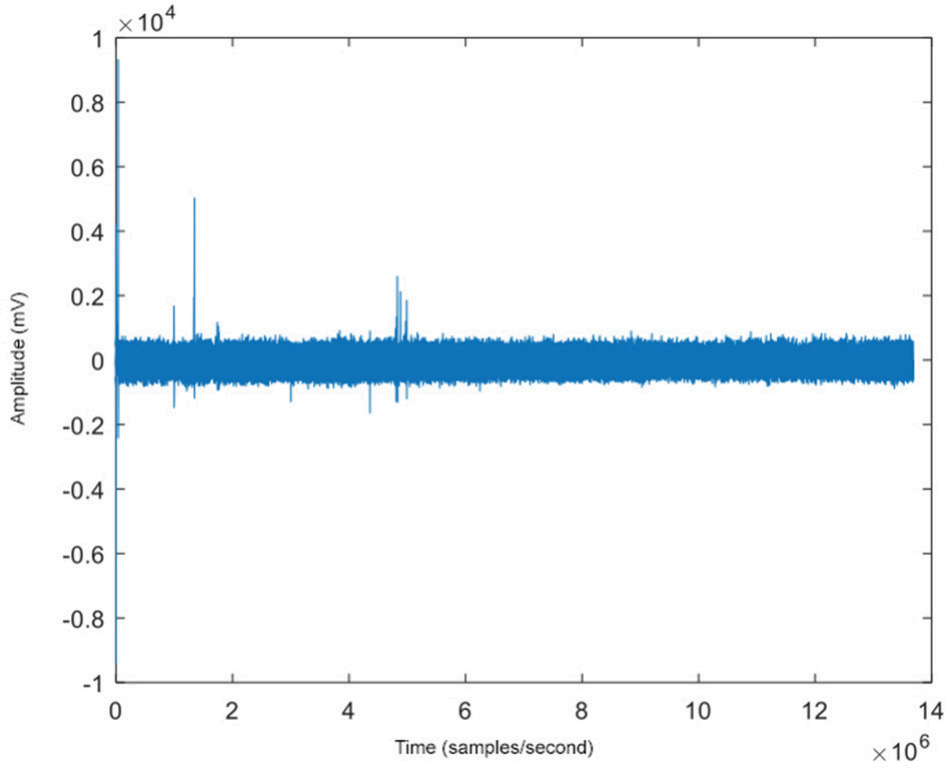
The raw EEG (with a sampling rate of 200 samples/second) of one of five channels for an example patient with some epileptiform spike activity seen at several time points.

**Figure 13:**
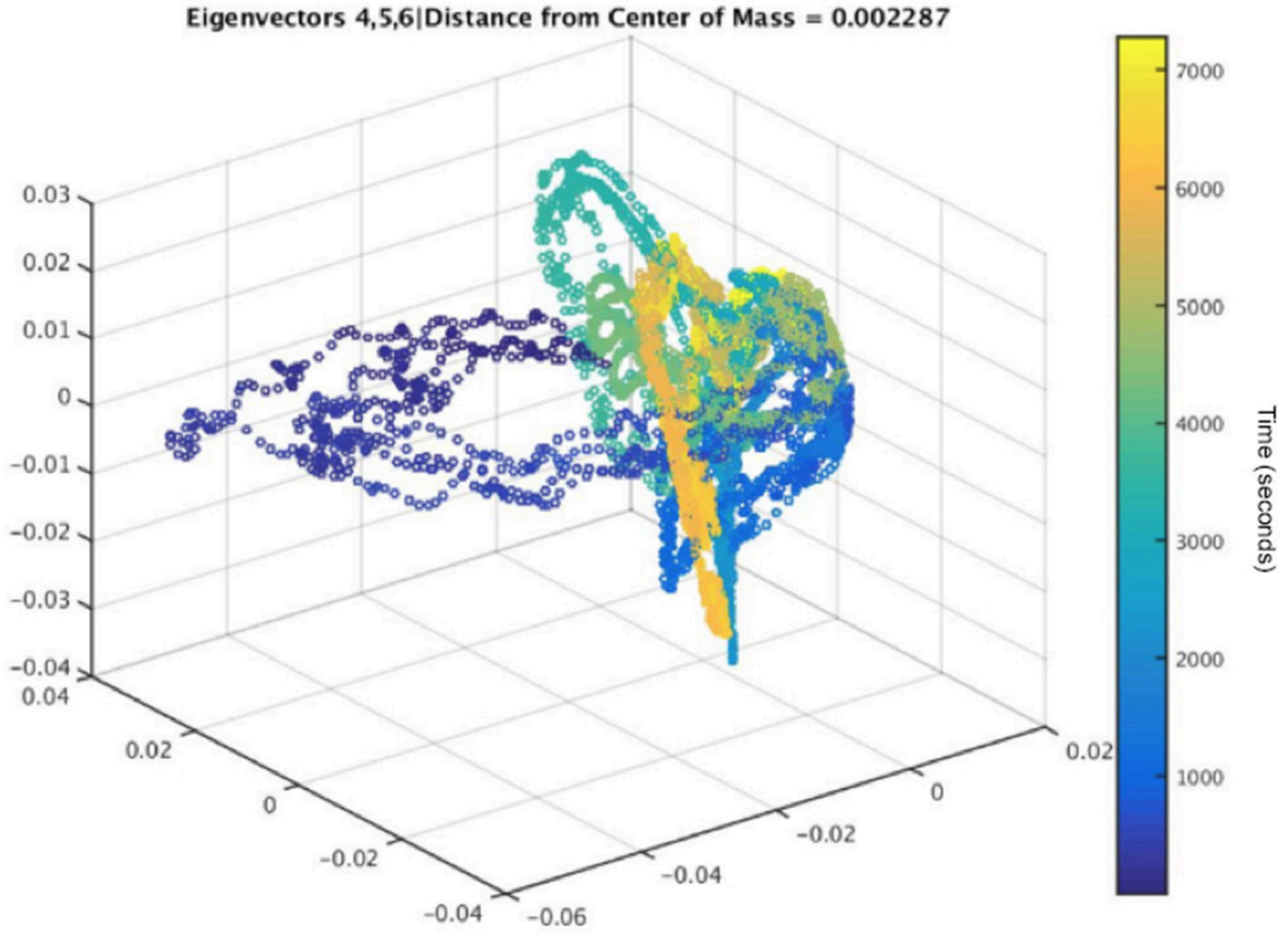
The embedding, corresponding to the data in Figure 12, with its average distance from the center of mass calculated for a 5-channel analysis. Color represents time, and the dark blue points show a clear separation from the other points, indicating a method to identify epileptiform activity.

The steps of this algorithm, UDCA, have been previously described (Duncan et al., 2018a); here we briefly explain the steps. The original, raw EEG data matrix (of any number of electrode contacts and any length of time), for example, Figure 12, is divided into smaller submatrices that are overlapped by 50% for smoothing purposes. First, the cross-correlation between segments is calculated to ensure minimal variance to ensure similar behavior between the channels that were being analyzed. Channels showing similar waveforms would be expected to have decreased covariance. This is applied to all channels used in the analysis (five channels in the example shown in Figure 13), after being split into submatrices. The limit is defined as the difference between the window size, the number of data points in the predefined submatrices, and the window length, the number of data points used to define the lag of the cross-correlation. Then the time-based covariance matrix is calculated from the covariance of the segment vectors. Singular value decomposition (SVD) is then performed on the covariance matrices. The Mahalanobis distance is applied to inverse covariance matrices that are computed using the SVD to identify outliers; the combination of the Mahalanobis distance and inverse covariance matrices has previously been shown to be a successful tool for denoising data (Talmon et al., 2012). The resulting matrices are constructed from the outputs of the SVD by taking the complex conjugate transpose of the product of the unitary matrix, the inverse of the diagonal matrix, and the other unitary matrix.

The next steps of the algorithm involve constructing the kernel, shown in Equation (3)

(4) $$\begin{array}{l} A=\text{exp}\left(\frac{-d}{4∙k_{e}}\right) \end{array}$$

where *d* is the Mahalanobis distance (Equation 5), and *k*_*e*_ is the Gaussian kernel (value set to 10, based on the spread of the original data points in the raw EEG data matrix) (Duncan and Strohmer, 2016).

(5) $$\begin{array}{l} d=\left[{\text{data}}_{M}-{\text{data}}_{m}\right]\cdot \;\overset{-1}{\underset{EEG}{\sum }}\cdot \left[{\text{data}}_{M}-{\text{data}}_{m}\right] \end{array}$$

in which data_M_ is the length of the i^th^ row from the metric data matrix, data_m_ is the i+1 row, and $\overset{-1}{\underset{EEG}{\sum }}\left[data_{M}-data_{m}\right]$ is the inverse covariance matrix (Duncan and Strohmer, 2016; Duncan et al., 2018a).

Construction of the reference kernel is shown below in Equation (5) using the inverse covariance and the natural extension of AA' (Duncan et al., 2013b, 2018a; Duncan and Strohmer, 2016):

(6) $$\begin{array}{l} W_{1}=A_{1}^{*}A_{1} \end{array}$$

in which *A*_1_ is the quotient of A divided element-wise by a repeat matrix of the square root of j_1_ with dimensions equal to that of the length of data_M_. W = A^*^A, in which A^*^ is the conjugate transpose, and W is the product of A and its conjugate transpose. Lastly, j_1_ = $\underset{i}{\sum }W_{1,i}$ (sum of the elements of W along its columns for row vector) (Duncan et al., 2013b; Duncan and Strohmer, 2016).

(7) $$\begin{array}{l} W_{2}=A_{2}^{*}A_{2} \end{array}$$

Additionally, Equation (6) is computed in the same manner as Equation (2), in which A_2_ (computed similarly to A_1_) with element-wise division by a repeat matrix of the square root of j_2_.

The computation of the eigenvectors Equation (7) is performed on W_2_, extracting the eigenvalues in a diagonal matrix V and the eigenvectors in a matrix E, corresponding to the eigenvalues, such that:

(8) $$\begin{array}{l} EV=W_{2}V \end{array}$$

The corresponding eigenvectors are then sorted in a descending order (E_srt_, V_srt_). Corresponding point clouds are calculated from Equation (8):

(9) $$\begin{array}{l} V_{clds}=DV_{srt} \end{array}$$

in which D is a sparse n x n matrix with the dimensions equal to the length of data_M_, with values consisting of the square root of one divided-by j_2_.

Extraction of the two largest eigenvectors was performed according to Equations (9, 10):

(10) $$\begin{array}{l} \varphi_{1}=V_{clds_{i,1}}\varphi_{2}=V_{clds_{i,2}} \end{array}$$

Computation of the extension utilized (Equation 11):

(11) $$\begin{array}{l} \omega =\underset{i}{\sum }A_{2_{i}} \end{array}$$

in which the column vector ω is the column-wise sum of A_2_.

Additionally, A_2_-norm (||*A*_2_||) is calculated by element-wise division of A_2_ by a repeat matrix consisting of values from ω, with dimensions equal to that of data_m_.

(12) $$\begin{array}{l} \hat{\psi }=\frac{||A_{2}||V_{srt_{i}\;}}{\sqrt{E_{srt_{i+1}}}} \end{array}$$

Furthermore, $\hat{\psi }$ (Equation 12), is calculated to be the product of ||*A*_2_|| and *V*_*sr*_*t*__*i*__divided element-wise by the square root of the i-th + 1 value of *E*_*srt*_.

Additionally, ψ (initialized as an empty array) is:

(13) $$\begin{array}{l} \psi_{i}=\psi \hat{\psi } \end{array}$$

Extended eigenvector extraction corresponding to the two largest eigenvalues (Equations 14, 15):

(14) $$\begin{array}{l} \psi_{1}=\;\psi_{i,1} \end{array}$$

(15) $$\begin{array}{l} \psi_{2}=\;\psi_{i,2} \end{array}$$

in which ψ_1_ and ψ_2_ are tabulated using all values from the rows and columns one and two, respectively.

#### Preliminary Results Using UDCA

All possible combinations of 3 eigenvectors are used to create the 3D embeddings. Three dimensions were chosen due to this number of dimensions being optimal for visualization, but any number can be chosen and then determined which number of dimensions results in the most important information about the underlying brain activity being extracted, depending on the original data. Embeddings that contained the first eigenvector were excluded due to the normalization that occurs as a result of the SVD analysis (Duncan and Strohmer, 2016). Furthermore, some preliminary results indicated that the embeddings that showed a more diffused spread of points with outliers could be used to indicate preseizure activity in the subject. The determination of the spread for each embedding was calculated by finding each embedded point's Euclidean distance from the center of mass of the embedded points. Embeddings with the largest mean Euclidean distance for each subject were used for preseizure activity evaluation. This method of determining the optimal embedding allows the algorithm to be automatic and unsupervised, but the algorithm can also be used in a semi-supervised manner as well.

The dark blue points in Figure 13 represent the time farthest from the seizure in the selected epoch, while the yellow points represent windows of time that are closest to the occurrence of the seizure. Figure 12 shows an example subject with EEG data from channel 4, in a 5-channel analysis, in which epileptiform spike activity is apparent at several initial time points. The outliers in the embedding shown could be used to correspond with several of the epileptiform spikes in the raw EEG data.

UDCA is a promising method that can be used to detect epileptiform activity that may be a predictor of post-traumatic epileptogenesis. Quantitatively, the evaluation of each embedding can be performed through a variety of methods, such as evaluating the diffusivity in the embedding by calculating the Euclidean distance of each point in the embedding to either the origin or the center of mass of all embedded points or by setting a threshold for the outlier points.

## Discussion

We have described some of our analytic tools, including lesion mapping, tractography, PVS analysis, and various types of EEG analysis, including spectral analysis, spike detection, mutual information, and Unsupervised Diffusion Component Analysis, that we are developing and using to analyze the rich, multimodal data from different sites that are collecting data for EpiBioS4Rx. Furthermore, the tools applied to imaging and EEG data are used for both human and animal data so that we can first analyze them separately and then compare the animal model to the human data to determine what translational components exist.

With tractography, we plan to explore the use of a study-specific template that may improve registration performance. We also plan to use the lesion mapping obtained from FLAIR to add lesion statistics to the array of obtained fiber bundle metrics. Based on our analysis of PVS, our results show that PVS may be a potential non-invasive neuroimaging biomarker of post-traumatic epileptogenesis. Moreover, PVS structural analysis combined with DTI analysis can help define the suspected seizure onset area. Ultimately, these results may be of benefit for the design of future clinical trials and for the evaluation of new possible therapeutic targets.

We plan to analyze the EEG using mutual information and compare those results with the resting state fMRI data to study networks in the brain, how they change over time, and how they differ between PTE and non-PTE. With UDCA, our goal is to apply advanced statistical tools to the results of the embeddings to reliably identify epileptiform and preseizure activity in the EEG of humans and rodents.

## Conclusions

As more data are collected in EpiBioS4Rx, we will continue to extract features from neuroimaging and electrophysiologic data as well as molecular, clinical, cognitive, and behavioral measures to identify candidate diagnostic biomarkers of epileptogenesis. When we apply these methods to new data, we will be able to modify and improve them so that they can be even more effective in our search for biomarkers of epileptogenesis after TBI. Our methods will be used to reveal processes, regions, and stages in epileptogenesis correlated with specific anatomical changes in imaging and changes in the electrical activity in the brain. Furthermore, our tools will allow us and other researchers to easily compare human and animal data to identify their similarities and differences. Innovative statistical techniques will be used to build models of epileptogenesis to predict the probability of developing epilepsy based on biomarker inputs.

## Author Contributions

DD took the lead in writing the manuscript with input from all authors, performed the EEG analysis, and developed one of the methods, UDCA. GB performed the PVS calculations, analysis, and interpretation. RC performed the tractography and diffusion MRI analysis as well as the interpretation. FS performed the lesion mapping analysis and interpretation. RG completed the literature review. AB assisted with the EEG analysis. PV collected the human data and assisted with questions relating to the human data. AP collected the rodent data and assisted with questions relating to the rodent data. ML supervised the PVS analysis. AT assisted with data storage issues, supervising all analysis, and directing the project with DD. All authors discussed the results, provided critical feedback, and contributed to the final manuscript.

### Conflict of Interest Statement

The authors declare that the research was conducted in the absence of any commercial or financial relationships that could be construed as a potential conflict of interest.
